# Noninvasive Testing for Diagnosis of Stable Coronary Artery Disease in the Elderly

**DOI:** 10.3390/ijms21176263

**Published:** 2020-08-29

**Authors:** Sergey G. Kozlov, Olga V. Chernova, Elena V. Gerasimova, Ekaterina A. Ivanova, Alexander N. Orekhov

**Affiliations:** 1 National Medical Research Centre of Cardiology of the Ministry of Health of the Russian Federation, 3-rd Cherepkovskaya str., 15a, Moscow 121552, Russia; bestofall@inbox.ru (S.G.K.); glazunova-23@mail.ru (O.V.C.); 2 V.A. Nasonova Institute of Rheumatology, 34A Kashirskoye Shosse, Moscow 115522, Russia; gerasimovaev@list.ru; 3 Institute for Atherosclerosis Research, Skolkovo Innovative Center, Moscow 121609, Russia; 4 Institute of Human Morphology, 3 Tsyurupa Street, Moscow 117418, Russia; 5 Laboratory of Angiopathology, Institute of General Pathology and Pathophysiology, 8, Baltiyskaya st., Moscow 125315, Russia

**Keywords:** coronary artery disease, older adults, exercise ECG, exercise stress echocardiography, coronary computed tomography angiography

## Abstract

Efficient diagnostic approaches to detect coronary artery disease (CAD) in elderly patients are necessary to ensure optimal and timely treatment. The population of suspected CAD patients older than 70 years is especially vulnerable and constantly growing. Finding the optimal diagnostic approach is challenging due to certain features of this population, such as high prevalence of comorbidities, existing contraindications to exercise tests or cognitive decline, which hinders correct assessment of the patient’s situation. Moreover, some symptoms of CAD can have variable significance in the elderly compared to younger adult groups. In this review, we present current recommendations of the United States (US) and European cardiologists’ associations and discuss their applicability for diagnostics in the elderly population. Exercise electrocardiogram (ECG) and exercise stress echocardiography (SE) tests are not feasible for a substantial proportion of elderly patients. Coronary computed tomography angiography (CTA) appears to be an attractive alternative for such patients, but is not universally applicable; for instance, it is problematic in patients with significant calcification of the vessels. Moreover, more studies are needed to compare the results delivered by CTA to those of other diagnostic methods. Future efforts should be focused on comparative studies to better understand the limits and advantages of different diagnostic methods and their combinations. It is possible that some of the currently used diagnostic criteria could be improved to better accommodate the needs of the elderly population.

## 1. Introduction

Demand for diagnostic methods of coronary artery disease (CAD) in the elderly is constantly growing, possibly due to increasing life expectancy, the growth of the elderly population and the direct link between the risk of CAD and older age. Diagnosis of CAD worldwide is currently based on protocols that were developed and revised over many years, and stipulate the diagnostic tests and medical imaging procedures to be implemented depending on each patient’s situation. Guidelines for diagnosis and management of stable CAD published by European and American cardiology associations are currently the most known and serve as benchmarks in different countries worldwide. While these guidelines are reliable for a large proportion of adult patients, it is possible that their applicability to the elderly population (individuals older than 70 years) could be further improved. Older adults often present with a set of clinical features that makes the use of standard diagnostic tests, such as exercise tests, challenging. Moreover, noninvasive imaging techniques that could be safely used in the elderly are sometimes not readily available in countries beyond Europe and North America, thereby increasing waiting time and associated costs. Imaging applicability can also be limited by certain morphological features that are more frequent in the elderly, such as pronounced blood vessel calcification. Therefore, the current diagnostic approach in older adults could possibly be further refined and improved. In this review, we discuss the current recommendations for the diagnosis and management of CAD published by European and American associations and applicability to elderly patients.

Despite the great progress made over the last decades, our understanding of the pathogenesis of atherosclerosis is far from complete, with some key questions remaining unanswered. For instance, factors that determine whether the pathological process leads to stable plaque formation or to plaque progression with severe consequences only started to be explored recently [1]. Detailed studies regarding atherosclerotic plaque progression are complicated by the fact that the mechanisms that are responsible for this process cannot be fully replicated in animal models [2]. Irreversible changes in the blood vessels accumulate with age, with growth of the arterial intima–media thickness due to the atherosclerotic process considered to be one of these. Atherosclerosis and associated CAD are traditionally listed among age-related diseases, although the presence of asymptomatic atherosclerosis in young adults is well-recognized [3]. Numerous pathophysiological studies focused on the cellular and metabolic features of atherosclerosis development in aging arteries. Among the key age-associated risk factors identified are chronic inflammation, accumulating mitochondrial dysfunction and oxidative stress, deficiency of stem cells that could replenish the damaged cellular components of the arterial wall and epigenetic alterations [4]. Older age is associated with changes in nutrient and lipid metabolism, as well as intracellular transport of nutrients and lipids, which favors intracellular lipid accumulation, foam cell formation and atherosclerosis development [5]. Moreover, vascular calcification is a well-recognized, age-associated risk factor. Atherosclerotic plaque calcification was found to be more pronounced in the elderly compared with younger patients [6]. Future studies should reveal links between aging and atherogenesis in more detail to help find points of therapeutic intervention.

Clinical manifestations of atherosclerosis and CAD in elderly patients are characterized by certain features that should be considered during diagnostic procedures. For instance, elderly patients with atypical angina and nonanginal pain are more likely to have obstructive CAD than younger patients presenting with the same symptoms [7]. Elderly patients that present with exercise-induced dyspnea also have an increased chance that their dyspnea is associated with angina pectoris [8]. Furthermore, multimorbidity, which complicates diagnostic procedures, is typical for elderly patients [9,10]. These patients have an increased likelihood of suffering from diseases other than CAD with overlapping symptoms that may shift the attention from less pronounced signs specific for CAD. Finally, age-associated cognitive impairment may hinder medical history taking.

A number of studies reported that episodes of asymptomatic CAD are more frequent in the elderly. One study that included 407 volunteers aged 40–96 years with no clinical manifestations of CAD revealed silent CAD episodes in 2% of subjects aged 50–60 years, and in 15% of subjects aged 90 years and older [11]. Prevalence of asymptomatic CAD can reach 40% among the elderly with no clinical history of the disease and 50% in patients with documented history of CAD [12]. A study by Aronow et al. included 915 men (mean age of 80 years) and 1874 women (mean age of 81 years) [13]. In this study, asymptomatic CAD was diagnosed in 6% of males and 5% of females with no history of cardiovascular disease, and in 34% of males and 33% of females with ischemic heart disease. Multivariate analysis identified the age of ≥74 years as an independent risk factor of asymptomatic CAD [14]. Some of the published studies of asymptomatic CAD delivered questionable results. Silent myocardial ischemia is defined as “objective documentation of myocardial ischemia in the absence of angina or angina equivalent” [15]. In many cases, exercise-induced ST-segment depression during continuous electrocardiogram (ECG) monitoring or exercise ECG testing is used for documentation of myocardial ischemia. However, the same symptoms may occur when obstructive CAD is not present. Many studies used exercise-induced transient myocardial perfusion defects for documentation of asymptomatic CAD, but such defects can also occur without obstructive CAD. Prevalence of asymptomatic CAD reported in some studies [12] was so high that it would necessitate noninvasive diagnostic procedures for any elderly patient, which is not in line with any of the published guidelines for stable CAD management.

Among elderly patients, up to 50% present with resting ECG abnormalities, including nonspecific ST–T changes [8]. High prevalence of arrhythmia and conduction defects [16] as well as nonspecific ST–T changes hinder ECG exercise testing for stable CAD diagnostic. Elderly patients are less fit in terms of physical exercise due to age-related changes and deconditioning, as well as musculoskeletal system, peripheral artery, pulmonary and neurological diseases. Reduced capacity for physical exercise could be misinterpreted as CAD manifestation and hamper ECG exercise testing. Moreover, elderly patients often present with elevated blood pressure, which may preclude reaching the diagnostic criteria in exercise tests due to blood pressure increase. Finally, elderly patients are more likely to experience anxiety before diagnostic tests.

## 2. Pretest Probability of Obstructive CAD in Elderly Patients with Suspected Stable CAD

**Figure 1:**
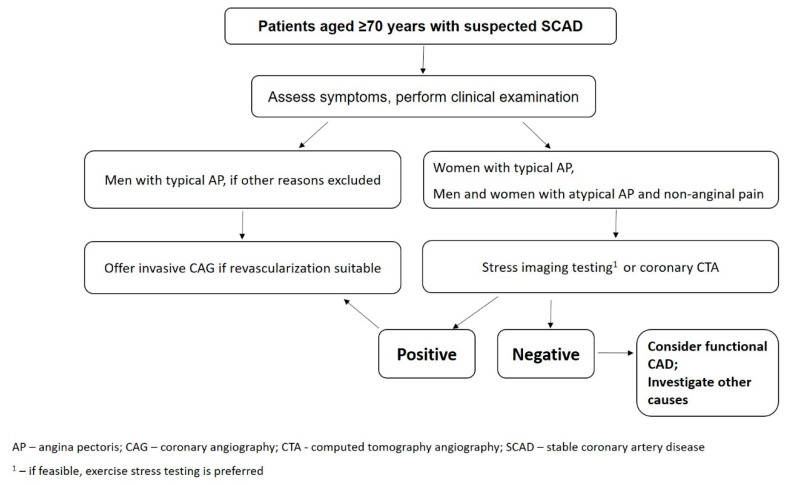
Diagnostic management of older adult patients with suspected SCAD.

Pretest probability (PTP) of obstructive CAD is evaluated during the first stages of stable CAD diagnostic, taking into account age, sex and chest pain complaints of the patient [17,18,19]. The outcome of PTP evaluation defines the following steps: Putting the patient under observation, noninvasive diagnostics, or invasive coronary angiography (CAG). Until recently, the most frequently used model for evaluation of PTP of obstructive CAD was that developed by Diamond and Forrester, published in 1979 [20]. This model is used in the current US recommendations for the diagnosis and management of patients with stable CAD [12]. In this publication, the table defining the criteria for PTP does not include patient’s age ≥70 years (Table 1). We also suggest a possible algorithm for diagnostic procedures for patients aged ≥70 years (Figure 1). European recommendations for the management of stable CAD published in 2013 use the model proposed by Genders et al., which is an update of the Diamond and Forrester model based on the results of a clinical study including 2260 patients, 570 of whom were older than 70 years [7,17]. In this model, values of PTP for patients older than 70 years were included. The study did not report the distribution of patients by sex or type of chest pain. Low (<15%) PTP does not require noninvasive diagnostic, while moderate PTP (15–85%) requires functional tests or noninvasive diagnostics of obstructive CAD. High PTP (>85%) establishes the CAD diagnosis and requires invasive CAG to assess the need for myocardial revascularization. All patients aged ≥70 years (except male patients with typical angina), regardless of the type of chest pain they experience, have a PTP requiring noninvasive diagnostic. Male patients aged ≥70 years with typical angina have a high PTP, which defines the CAD diagnosis.

European recommendations for the diagnosis and management of chronic coronary syndromes were published in 2019 [19]. This publication contains a table with PTP values that are much lower than in the previous 2013 edition. The updated values of PTP were based on a meta-analysis of three clinical studies [21], of which two [22,23] included CAD diagnosis based on coronary computed tomography angiography (CTA). In the third study, only 17% of patients underwent invasive CAG [24], raising the question of justification of the replacement of invasive CAG (the gold standard of CAD diagnostic) with another diagnostic test. Published recommendations explain that PTP of CAD depends on the prevalence of the disease in the studied population. It can therefore be speculated that CAD prevalence decreased since the last edition of recommendations in 2013. However, the diagnostic process should take into account the CAD prevalence not in a general population, but in patient groups stratified by age, sex and presence of chest pain. The authors did not provide any explanation regarding the decreased prevalence of CAD in the European population of patients of both sexes with typical angina. Current recommendations stipulate that in patients with PTP <5%, further diagnostic tests should be performed only if valid reasons are present. In patients with PTP of 5–15%, noninvasive diagnostic tests are indicated if certain risk factors are present. In case of PTP >15%, noninvasive diagnostic tests are indicated for all patients. According to these recommendations, all patients aged ≥70 years (except for women with nonanginal pain or dyspnea) meet the criteria for indication of noninvasive diagnostic tests for stable CAD (Table 1). Women aged ≥70 years with nonanginal pain should be indicated for noninvasive diagnostic tests only if risk factors are present. According to European recommendations issued in 2019, only 52% of men and 27% of women aged ≥70 years with typical angina were estimated to have obstructive CAD. The authors of these recommendations did not provide an explanation for the presence of typical angina in the remaining 73% of women and 48% of men.

Besides the type of chest pain, age and sex, some models (e.g., the CAD consortium clinical model, UK NICE model, Duke Clinical Score) take into account CAD risk factors to estimate the PTP. The rationale for this approach for patients aged ≥70 years is unclear. According to the results of several studies, the presence of CAD risk factors does not contribute to the likelihood of having CAD in this group of patients [25,26,27]. Although European guidelines of 2019 recommend considering the presence of main CAD risk factors for PTP assessment, they do not detail the values of the probability increase attributed to each of these factors [19].

PTP can be estimated based on the presence of resting ECG abnormalities. According to some authors, the presence of nonspecific ST–T changes in patients with suspected stable CAD increases the PTP [18,19]. Abnormalities in the resting ECG in elderly patients are associated with increased risk of adverse cardiac events [8]. However, no studies exist regarding the importance of nonspecific ST–T changes in addition to chest pain and sex when assessing PTP in patients with suspected stable CAD.

## 3. ECG Exercise Testing for Diagnosis of Stable CAD in the Elderly

Among noninvasive tests for diagnosis of stable CAD, ECG exercise testing remains the most frequently used due to availability, simplicity and lower cost compared to imaging methods. Such testing is recommended for patients capable of physical exercise. According to US recommendations, ECG exercise testing is indicated for patients with moderate PTP with no ECG abnormalities that could interfere with test result interpretation and who are capable of physical exercise (class of recommendations I, level of evidence A) [18]. However, the recommendations do not stipulate the values of moderate PTP. The Canadian recommendations for diagnosis and treatment of CAD include the same table for PTP evaluation as the US recommendations [28]. In the footnotes, it is specified that PTP <10% is low, 10–90% is moderate and >90% is high. European recommendations published in 2019 recommend ECG exercise testing as an alternative when imaging diagnostic methods are not feasible (class of recommendations IIb, level of evidence B) [19].

Elderly patients are often excluded from clinical studies that evaluate the accuracy of ECG exercise testing for diagnosis of stable CAD. Extrapolation of the results of such studies to the elderly population is therefore not appropriate. Only a few studies including elderly patients are currently published, and the information they deliver is limited [29,30,31]. These studies often included low numbers of patients, recruited patients with documented CAD and did not exclude patients for whom ECG exercise testing was not indicated due to high PTP. Repeated studies could include ECG exercise testing with different methodologies, which could be combined with other results, allowing results of ECG exercise testing results to be interpreted even when the diagnostic criteria were not met. Studies that assessed specificity and sensitivity of ECG exercise treadmill testing for diagnosis of CAD in the elderly had serious methodological limitations [30,32]. In the study of Newman and Phillips, sensitivity and specificity of the test in patients aged ≥65 years were 85% and 56%, respectively [32]. Since elderly patients present with severe CAD more frequently than younger age groups, the authors suggested that ECG exercise testing may be more sensitive and moderately less specific in elderly patients [33].

Meta-analyses of studies including patients regardless of age reported that the sensitivity and specificity of ECG exercise testing for diagnosis of stable CAD were 58–68% and 62–77%, respectively [34,35]. European recommendations published in 2013 reported sensitivity of 45–50% and specificity of 85–90% [18]. Comparison of different studies reveals large differences in sensitivity and specificity parameters of the same ECG exercise testing. A meta-analysis performed by Detrano et al. reported sensitivity values ranging from 40% to 90% and specificity values from 50% to 100% [35]. Such differences may be explained by the heterogeneity of the patient populations included in these studies according to such parameters as mean age, sex and pain complaints [36]. Different studies used different exercise protocols, positive and negative results criteria and CAD criteria. Differences in CAD prevalence among patients from different studies means that comparison of the results of these studies would be inappropriate, thereby hindering evaluation of the tests’ diagnostic accuracy levels. The positive predictive value (PPV) in CAD diagnostics tends to be higher in elderly patients. At the same time, the negative predictive value (NPV) tends to be lower in this age group compared to younger age groups. This is due to the higher prevalence of CAD in older people. This tendency was demonstrated in the study of Levisman et al., who showed a 36% increase in treadmill test PPV in women aged 35–50 years and 68% in women older than 65 years [37]. Studies of the positive and negative likelihood ratio (LR+ and LR−) of PPV and NPV of ECG exercise testing in diagnosis of CAD was not performed in elderly patients. A meta-analysis showed that the LR+ and LR- of treadmill testing in patients from all age groups were 3.57 and 0.38, respectively, while corresponding values for bicycle exercise testing were 2.94 and 0.4, respectively [38]. These results indicated that the difference between pre- and post-test likelihood of CAD were rather small. A positive treadmill test result increased the likelihood of having CAD from 57% to 80%, while a negative result reduced it to 28%. A positive bicycle exercise test result increased the likelihood of having CAD from 56% to 80%, while a negative result reduced it to 35%. Changes in post-test probability were too small to cause the baseline intermediate probability to become very high (>85%) or very low (<15%).

## 4. Exercise Stress Echocardiography for Diagnosis of Stable CAD in the Elderly

Exercise stress echocardiography (SE) is the most attractive CAD diagnostic method in the elderly. Unlike myocardial perfusion scintigraphy, this method does not require irradiation and it is characterized by better availability and lower cost than other diagnostic methods. According to US recommendations for the diagnosis and management of patients with stable CAD, exercise SE is indicated for patients with intermediate PTP that are capable of physical exercise and show ECG abnormalities that hinder interpretation during exercise (class of recommendation I, level of evidence B) [18]. European recommendations issued in 2019 formulate indications for visualization methods during exercise tests less precisely than the previous issue of 2013 [19]. According to current recommendations, such tests are indicated for patients with symptoms, for whom CAD cannot be excluded based on clinical evaluation (class of recommendations I, level of evidence B), without stipulating procedures for the latter. In the presence of symptoms indicative of CAD, if clinical evaluation excludes other reasons for these symptoms, any patient can be suspected of having CAD with some kind of probability. According to the recommendations, the choice of noninvasive diagnostic method should depend on the clinical likelihood of CAD (class of recommendations I, level of evidence C). This term is used in place of PTP. Assessment of clinical likelihood takes into account the presence of CAD risk factors along with the pain complaints, age and sex of the patient. However, it is unclear from the recommendations the values attributed to each risk factor and therefore what contribution they make to the final result.

Few studies assessed the accuracy of exercise SE for detecting stable CAD in the elderly. The study by Gurunathan et al. combined the results of patients with documented CAD that followed different exercise methods [39]. Meta-analyses of studies that included patients from all age groups reported s sensitivity of exercise SE of 79–85% and s specificity of 77–89% [34,40,41,42,43]. No studies evaluated LR+ and LR− of exercise SE in elderly patients. Meta-analyses of studies including patients from all age groups reported LR+ and LR− values of 11.34 and 0.17 for bicycle exercise testing and 7.94 and 0.19 for treadmill testing [38]. A positive bicycle exercise SE result increased the likelihood of CAD from 49% to 92%, while a negative result reduced it to 16%. A positive result of treadmill exercise SE increased the likelihood of CAD from 55% to 89%, while a negative result reduced it to 19%. The results of studies including patients from all age groups indicated the sensitivity and specificity of exercise SE, as well as higher LR+ and lower LR− values compared to ECG exercise testing. However, it remains unknown whether the capabilities of these two methods for CAD diagnosis in the elderly are significantly different.

## 5. Coronary Computed Tomography Angiography for Diagnosis of Obstructive CAD in the Elderly

Coronary computed tomography angiography (CTA) is being used more and more frequently for CAD diagnostics thanks to increasing availability in hospitals and accumulating experience in using this method. According to the US recommendations, CTA is indicated for patients that are capable of physical exercise and have intermediate PTP (class of recommendations IIb, level of evidence B) [18]. Coronary CTA is also suitable for patients that are not capable of physical exercise and have low or intermediate PTP (class of recommendations IIa, level of evidence B), and patients with intermediate PTP that fail to reach the diagnostic limits of the exercise test (class of recommendations IIa, level of evidence C). The UK NICE guidelines issued in 2016 position coronary CTA as a first-line choice for diagnosis of stable CAD in patients with typical or atypical angina, as well as in patients with nonanginal chest pain and ECG abnormalities (ST–T changes and/or Q waves) [44]. Use of imaging-based methods is recommended in cases where coronary CTA would be noninformative or when obstructive CAD is doubtful based on functionality. These recommendations triggered vivid discussion [45,46,47]. One of the arguments for using CTA as a first-line choice in diagnostics was the lower frequency of myocardial infarction in patients who underwent coronary CTA compared to patients who only passed functional tests [48]. Another argument was the higher efficacy of coronary CTA for revealing obstructive CAD compared to functional tests [49]. According to European recommendations issued in 2019, if coronary CTA and exercise imaging testing are equally possible for a given patient, both diagnostic methods can be regarded as first-line choices [19].

Few studies using coronary CTA for diagnosis of stable CAD in the elderly exist in the literature [50,51,52,53,54]. A meta-analysis of 5332 patients, including 462 patients aged >75 years, reported a sensitivity of coronary CTA for diagnostic of obstructive CAD of 94% and a specificity of 77%, alongside a PPV of 83%, NPV of 91%, LR+ of 4.1 and LR− of 0.08 [54]. The authors revealed reduced efficacy of coronary CTA for the diagnosis of obstructive coronary atherosclerosis in patients aged >75 years compared to other age groups.

Diagnostic accuracy of coronary CTA was evaluated in several studies that included patients regardless of age group [55,56,57,58]. A study by Meijboom et al. included 254 patients, 105 with high (≥71%), 83 with intermediate (31–70%) and 66 with low (≤30%) PTP [55]. Coronary CTA sensitivity values were 98%, 100% and 100% in patients with high, intermediate and low PTP, respectively, with specificity values of 74%, 84% and 93%, respectively, PPV values of 93%, 80% and 75%, respectively, and NPV values of 89%, 100% and 100%, respectively. The LR+ values were 3.74, 6.38 and 13.5, respectively, and the LR− values were 0.03, 0 and 0, respectively. A positive coronary CTA result increased the likelihood of having CAD to 96%, 88% and 68%, respectively, while a negative result reduced it to 17%, 0% and 0%, respectively. The authors concluded that coronary CTA for diagnostic purposes was efficient in patients with intermediate and low PTP, but not in patients with high PTP. A study by van Werkhoven et al. included 61 patients with intermediate (13–87%) PTP [56]. In this study, the sensitivity of coronary CTA was 100%, the specificity was 89%, the PPV was 76%, the NPV was 100%, the LR+ was 9.1 and the LR− was 0. The ACCURACY study reported a sensitivity of 95%, a specificity of 83%, a PPV of 64% and an NPV of 99%. LR+ and LR− values were 5.56 and 0.06, respectively [58]. A meta-analysis of 18 studies reported a sensitivity of 98%, a specificity of 82%, a PPV of 91% and an NPV of 99% [59]. Another meta-analysis including 65 studies reported a CTA sensitivity of 95% and a specificity of 79% [54]. The efficacy of CTA in CAD diagnostics was not dependent on the type of chest pain. CTA was shown to be most suitable for patients with a PTP of 7–67%. A CTA of more than 64 slices was considered to be more efficient than CTA using a smaller number of slices. The authors reported that coronary CTA was less efficient for CAD diagnosis in women compared to men. Other studies, however, reported equal diagnostic accuracy of CTA in women and men with low and intermediate PTP [60].

There are no currently published studies showing that coronary CTA is more or less suitable than exercise SE for CAD diagnosis in the elderly. While exercise SE has a lower cost and higher availability, coronary CTA is more sensitive, has a greater PPV and a lower LR−, as demonstrated in studies including patients from all age groups. Differences in specificity, PPV and LR+ were not always visible. An important advantage of exercise SE over coronary CTA is the possibility to assess functionality. Moreover, it does not require using a contrasting product. Several studies compared the incidence of adverse cardiovascular events in patients who underwent exercise SE or coronary CTA [61,62]. The PROMISE study included 10,003 patients without previously documented CAD. After two years, the incidence of adverse cardiovascular events in 4996 patients who underwent coronary CTA was 3.3%, which was not different from the corresponding incidence among the 5007 patients who underwent functional tests (myocardial perfusion scintigraphy in 68% of cases, exercise SE in 22% and ECG exercise testing in 10%), in whom the incidence of adverse cardiovascular events was 3% [61]. Meta-analysis of the results of 13 studies including 10,315 patients showed that coronary CTA was associated with a reduced incidence of myocardial infarction in comparison to functional tests [48].

Exercise tests in elderly patients are often not feasible due to deconditioning and the presence of comorbidities, while pharmacological testing may be contraindicated [63]. In such situations, a decision is made regarding whether to use coronary CTA to assess the necessity of invasive CAG or to use invasive CAG immediately. In the first case, the patient receives a higher radiation dose and higher amount of contrasting product, which can increase the risk of nephropathy. This approach is also more expensive. In the second case, the risk of CAG-related complications increases, and this test is often poorly tolerated by the elderly patients. No studies as yet have been conducted to evaluate the advantages of both of these approaches. A randomized CONSERVE study, which included patients with suspected CAD regardless of age, showed that coronary CTA was associated with reduced frequency of CAG that did not reveal CAD and reduced cost of the diagnostic procedures [64].

Coronary CTA in elderly patients is often hindered by a pronounced calcification of coronary arteries [65]. Another problem is the necessity of breath-holding during the examination. This latter problem can currently be solved by the appearance of multislice/multidector/multichannel CT scanners, which reduce imaging time and are suitable for all patients. In parallel, contrasting agents used for computed tomography have also evolved; while early methods used iodine-containing contrasting reagents associated with adverse reactions, particularly in the elderly, modern, nonionic reagents reduce the risk of adverse events risk.

## 6. Conclusions

Diagnosing stable CAD in elderly patients has its specificities. Most patients, regardless of chest pain complaints and sex, have a PTP that requires noninvasive stable CAD diagnostics. Studies assessing the diagnostic accuracy of ECG exercise testing and exercise SE for stable CAD detection in the elderly do not provide sufficient information. No data are available to evaluate the differences in diagnostic accuracy levels of different methods in elderly patients. If both exercise SE and coronary CTA are feasible for a given patient, there are no available data to inform clinicians in terms of making this choice. However, exercise tests are often not feasible in the elderly because of deconditioning or the presence of comorbidities. In such cases, no data are available to support the choice of whether or not to perform coronary CTA prior to coronary angiography. In this work, we summarized the existing recommendations and literature data and proposed a possible algorithm for the diagnostic procedures of stable CAD in the elderly population based on this information.

## Figures and Tables

**Table 1:** Comparative summary of diagnostic method choices of European, United States (US) and Canadian guidelines.

	EU	US	Canada
Document	2019 ESC Guidelines for the diagnosis and management of chronic coronary syndromes [19].	2012ACCF/AHA/ACP/AATS/PCNA/SCAI/STS Guideline for the diagnosis and management of patients with stable ischemic heart disease [18].	Canadian Cardiovascular Society Guidelines for the diagnosis and management of stable ischemic heart disease [28].
Year	2019	2012	2014
Definition of PTP of obstructive CAD	All groups of patients aged ≥70 years have PTP >15%, which makes them eligible for noninvasive diagnostic tests (except for women with nonanginal pain [10%] or dyspnea as their primary symptom [12%]).	Age ≥70 years not taken into consideration.	Age ≥70 years not taken into consideration.
**Choice of Diagnostic Method**			
Exercise-ECG	Exercise ECG recommended as an alternative when imaging diagnostic methods are not feasible.	Exercise ECG indicated for patients with moderate PTP that: - Have an interpretable resting ECG; - Are capable of physical exercise.	Exercise ECG indicated for patients with moderate PTP (10–90%) that: - Have an interpretable resting ECG; - Are capable of physical exercise.
Exercise-SE	Exercise SE indicated for patients with symptoms for whom CAD cannot be excluded based on clinical evaluation.	Exercise SE indicated for patients with intermediate PTP that are capable of physical exercise and have ECG abnormalities that hinder interpretation.	Exercise SE recommended as initial test in patients capable of physical exercise and with ECG abnormalities that hinder interpretation.
Coronary CTA	Coronary CTA is the preferred test in patients with a lower range of clinical likelihood of CAD, no previous diagnosis of CAD and characteristics associated with a high likelihood of good image quality.	Coronary CTA indicated for patients that: - Have intermediate PTP and are capable of exercise; - Have low or intermediate PTP and are not capable of physical exercise; - Have intermediate PTP and fail to reach the diagnostic limits of the exercise test.	Coronary CTA is most appropriate for patients with a lower range of PTP or are in the intermediate risk category for CAD.

CAD, coronary artery disease; CTA, computed tomography angiography; ECG, electrocardiogram; PTP, pretest probability/likelihood of CAD; SE, stress echocardiography.
